# Supplementary figures and images for: The pro-resolving lipid mediator Maresin 1 ameliorates pain responses and neuroinflammation in the spared nerve injury-induced neuropathic pain: A study in male and female mice
Source: PLoS One. 2023 Jun 22;18(6):e0287392. doi: 10.1371/journal.pone.0287392 (PMC10286986; doi:10.1371/journal.pone.0287392)

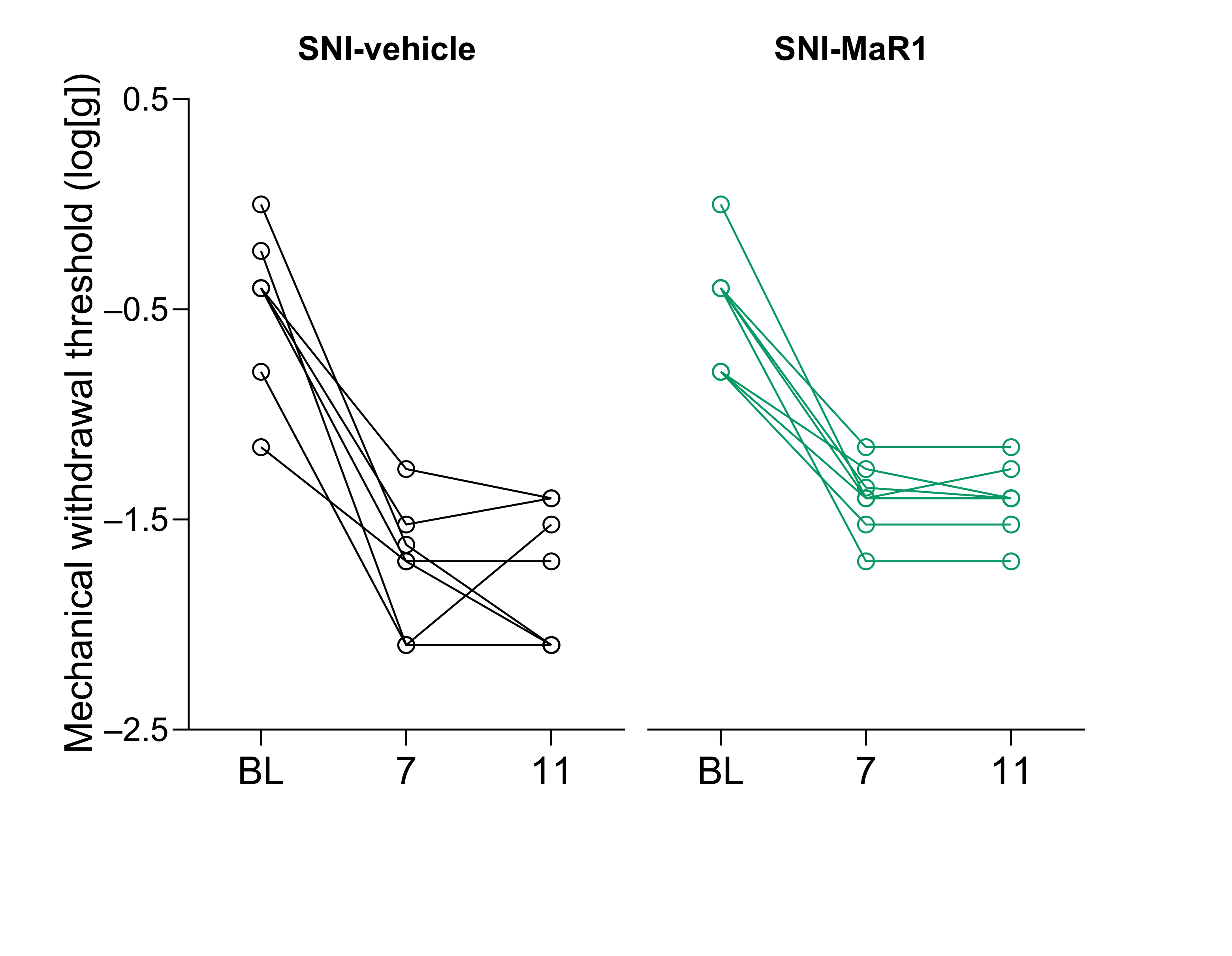

Supplement: S1 Fig — SNI-veh, n = 7; SNI-MaR1, n = 8. (TIF) [file pone.0287392.s001.tif]

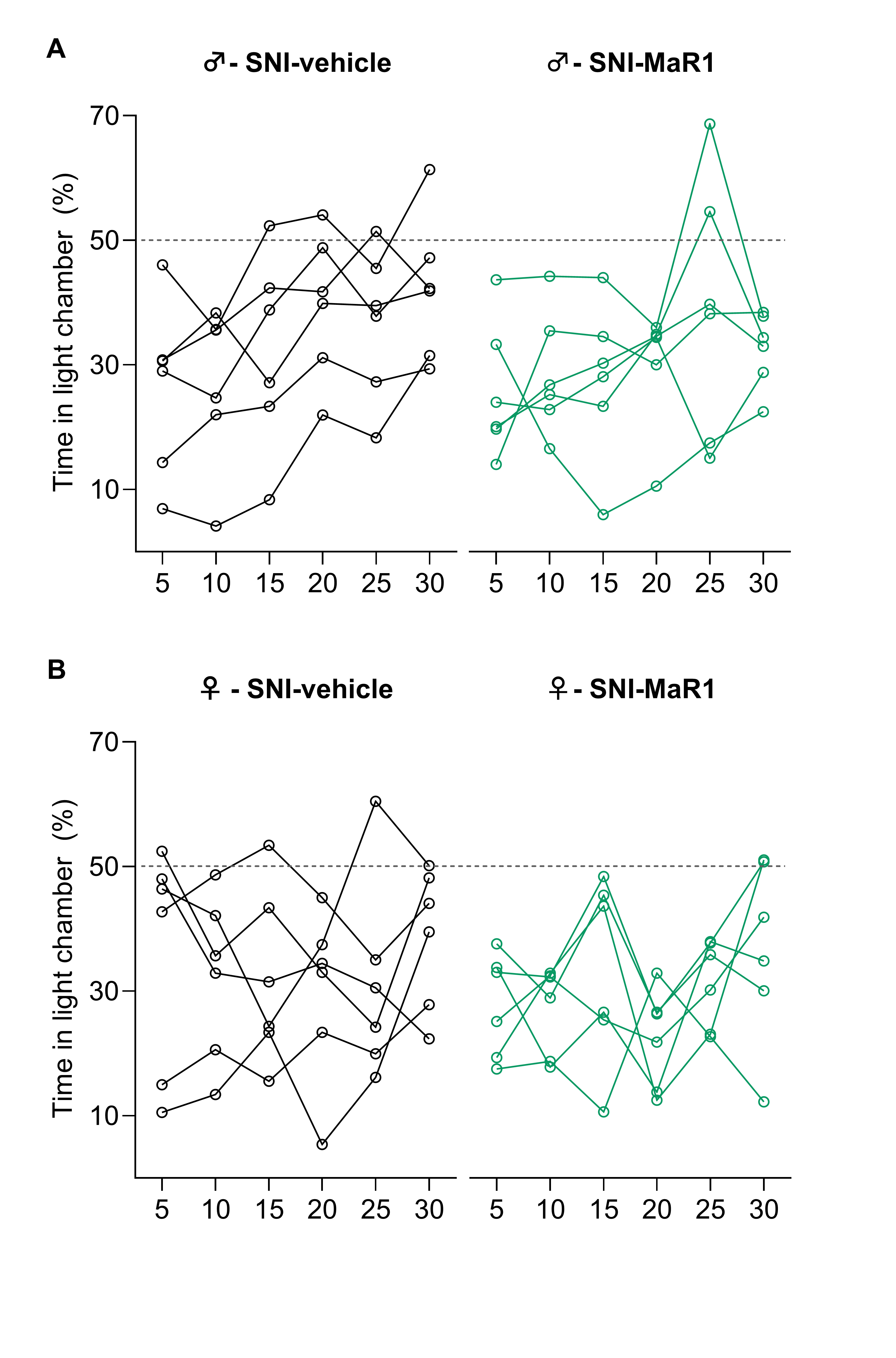

Supplement: S2 Fig — Individual behavior of male (A) and female (B) mice throughout the PEAP test period, on day 11 after spared nerve injury (SNI), presented as time spent in the light chamber (%) in time bins of 5 min throughout the 30-min test period. ♂-SNI-veh, n = 6; ♂-SNI-MaR1, n = 6; ♀-SNI-veh, n = 6; ♀-SNI-MaR1, n = 6. (TIF) [file pone.0287392.s002.tif]

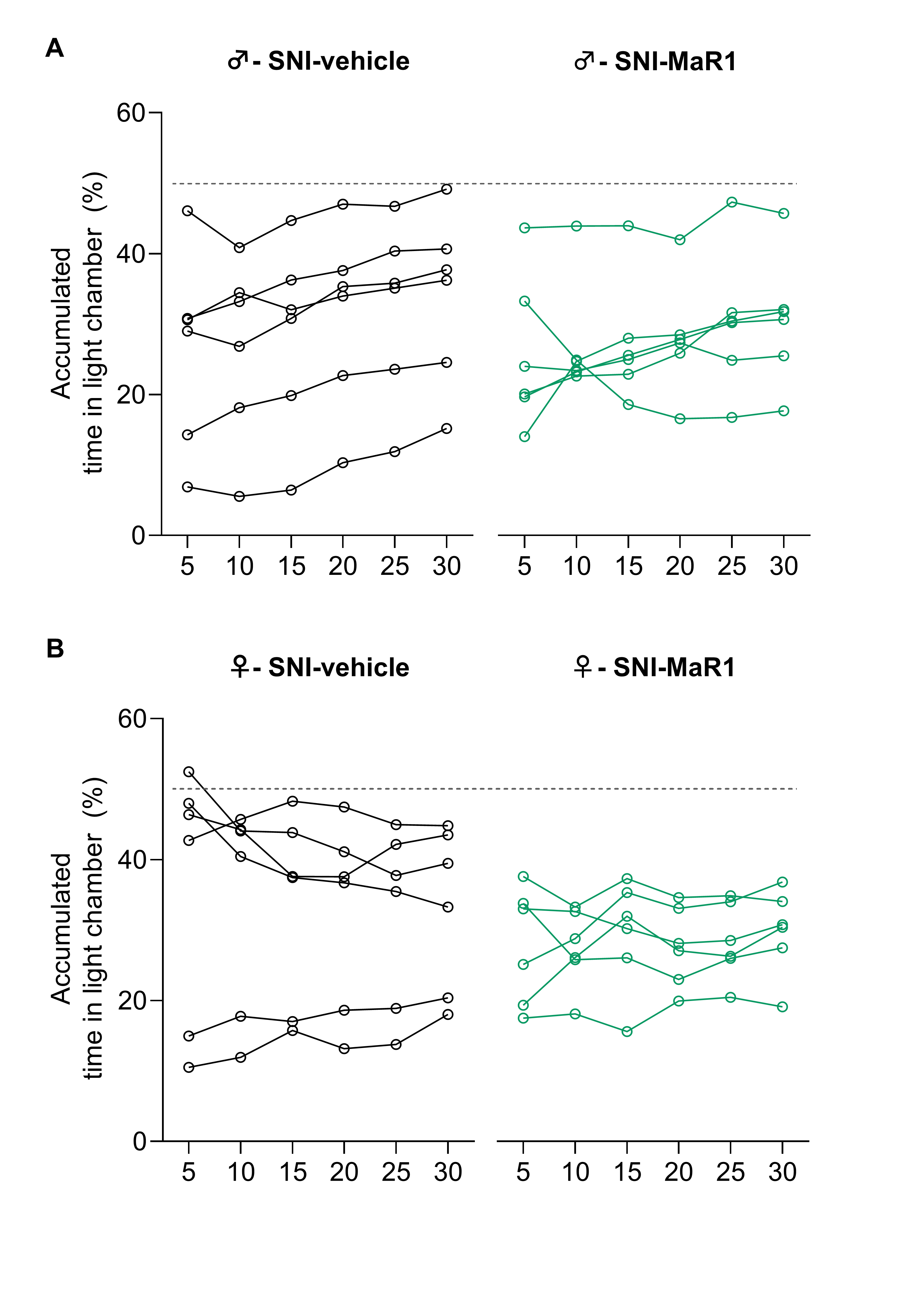

Supplement: S3 Fig — Individual behavior of male (A) and female (B) mice throughout the PEAP test period, on day 11 after spared nerve injury (SNI), presented as accumulated time spent in the light chamber (%) in time bins of 5 min. ♂-SNI-veh, n = 6; ♂-SNI-MaR1, n = 6; ♀-SNI-veh, n = 6; ♀-SNI-MaR1, n = 6. (TIF) [file pone.0287392.s003.tif]
