# Supplementary material for: The pro-resolving lipid mediator Maresin 1 ameliorates pain responses and neuroinflammation in the spared nerve injury-induced neuropathic pain: A study in male and female mice
Source: PLoS One. 2023 Jun 22;18(6):e0287392. doi: 10.1371/journal.pone.0287392 (PMC10286986; doi:10.1371/journal.pone.0287392)
Supplement: Attachment — Submitted filename: PlosOne_MaR1_ResponseReviewers.docx [file pone.0287392.s005.docx]

**Responses to the Reviewers**

We thank the reviewers for their valuable time and thoughtful feedback. Please find below our point-to-point response. Reviewers’ comments are shown in “*italics*”. For editor’s and reviewers’ convenience, both the complete replies to reviewers and a **track-changes version of the manuscript** have been uploaded. In the replies to reviewers, mentions to “line numbers” refer to the final manuscript, rather than the “track-changes version of the manuscript”.

**Reviewer #1:**

*The authors described the possible therapeutic potential of maresin-1 in spared nerve injury-induced neuropathic pain. I have one concern. In figure 5, please set the non-SNI control group to see if SNI treatment itself significantly increases these inflammatory cytokines, because previous many studies showed that maresin-1 suppresses the production of these representative inflammatory cytokines.*

Several studies have assessed the impact of peripheral nerve injury on spinal inflammatory cytokines. In general, IL-1β and IL-6 have been reported to be increased [Austin *et al*., 2010; Zhou *et al*., 2016], with IL-6 induction occurring slightly after IL-1β [Lee *et al*., 2004], although Martucci *et al.* have reported no changes in spinal IL-6 expression in male mice with peripheral nerve injury [Martucci *et al*., 2008]. Increased spinal M-CSF mRNA values have been reported in rats between days 2 and 14 post-SNI [Okubo *et al*., 2016]. Previous data from our group (obtained in a pilot experiment) on these inflammatory cytokines in SNI *versus* sham mice on day 11 post-injury has shown higher IL-1β concentration in both male and female SNI animals, higher IL-6 in SNI females (but not SNI males), higher M-CSF in SNI males (but not SNI females), and no differences in IL-10, in either sex.

However, considering that, in this work, our primary aim was to evaluate whether oral MaR1 treatment could modulate neuroinflammation in the SNI mouse model, our experimental design and planned statistical analysis only included the comparison between SNI males and females treated with vehicle or MaR1. Considering the Reviewer’s concern about the (widespread?) suppressive effects of MaR1 on these inflammatory cytokines, our results do not support that hypothesis. In fact, since we performed a Milliplex assay, all four cytokines were measured simultaneously in the same samples, and we have been able to detect an increase in M-CSF concentrations in MaR1-treated SNI males. Furthermore, our results on IL-10 (no differences found) are in agreement with previous studies that have tested MaR1 treatments on NP rodent models [Francos-Quijorna *et al*., 2017; Wei *et al*., 2022]. Nevertheless, we cannot exclude that subtle differences in IL-1β and IL-6 values might not have been detected due to the fact that we have used the whole spinal cord segment (some SNI-induced events may be predominantly localized in restricted areas). We now explicitly acknowledge this possibility in the section *On the neuroinflammatory mechanisms underlying MaR1 actions* (lines 632-634).

Austin PJ, Moalem-Taylor G. The neuro-immune balance in neuropathic pain: involvement of inflammatory immune cells, immune-like glial cells and cytokines. J Neuroimmunol. 2010;229(1-2):26-50. doi:10.1016/j.jneuroim.2010.08.013

Francos-Quijorna I, Santos-Nogueira E, Gronert K, et al. Maresin 1 Promotes Inflammatory Resolution, Neuroprotection, and Functional Neurological Recovery After Spinal Cord Injury. J Neurosci. 2017;37(48):11731-11743. doi:10.1523/JNEUROSCI.1395-17.2017

Lee HL, Lee KM, Son SJ, Hwang SH, Cho HJ. Temporal expression of cytokines and their receptors mRNAs in a neuropathic pain model. Neuroreport. 2004;15(18):2807-2811.

Martucci C, Trovato AE, Costa B, et al. The purinergic antagonist PPADS reduces pain related behaviours and interleukin-1 beta, interleukin-6, iNOS and nNOS overproduction in central and peripheral nervous system after peripheral neuropathy in mice. Pain. 2008;137(1):81-95. doi:10.1016/j.pain.2007.08.017

Okubo M, Yamanaka H, Kobayashi K, et al. Macrophage-Colony Stimulating Factor Derived from Injured Primary Afferent Induces Proliferation of Spinal Microglia and Neuropathic Pain in Rats. PLoS One. 2016;11(4):e0153375. doi:10.1371/journal.pone.0153375

Wei J, Su W, Zhao Y, et al. Maresin 1 promotes nerve regeneration and alleviates neuropathic pain after nerve injury. J Neuroinflammation. 2022;19(1):32. doi:10.1186/s12974-022-02405-1

Zhou YQ, Liu Z, Liu ZH, et al. Interleukin-6: an emerging regulator of pathological pain. J Neuroinflammation. 2016;13(1):141. doi:10.1186/s12974-016-0607-6

**Reviewer #2**:

*In this work, Teixeira-Santos and colleagues demonstrated that oral treatment with MaR1 is effective at reducing SNI-induced pain in rats. Novelty is the demonstration that voluntary intake of MaR1 in jam for 3 days is enough to reduce pain and mild sex-specific effect during escape/avoidance behaviors in MaR1-treated animals. The study is interesting, however, there are points that should be addressed:*

*1) How did Author monitor jam intake? Do all the animals ingest the whole 60 uL? Do Authors see any difference in intake for the vehicle- vs MaR1-containing jam? Would Authors expect gain weight upon chronic treatment?*

- Jam intake was monitored by continuous observation of mice behavior in their individual (transparent) cages, in order to confirm the full ingestion of the jam *plus* drug/vehicle mixture. Furthermore, Petri dishes (in which the mixture was placed and presented to mice) were visually inspected at the end.

- All animals completely ingested the jam mixture within a 5-min interval.

- No differences were observed in the intake of vehicle- or MaR1-containing jam. In fact, we have analyzed the latency time to ingest the jam and the jam *plus* admixed drug/vehicle, and have concluded that the addition of drug did not induce differences between groups.

- The caloric content of the strawberry jam that we have used was 196.2 kcal per 100 g. Therefore, and taking into consideration that the average daily consumption of feed for an adult 25 g mouse is 3-5 g (data from the Animal Care and Use Committee from Johns Hopkins University), we have estimated that the caloric contribution of the 60 µL of jam amounts for approximately 0.17 kcal, which corresponds to between 1 and 1.7% of the average daily caloric intake (for a 25 g mouse). Therefore, we expect neither significant weight gain nor diet changes with impact in our experimental model. Nevertheless, the suitability of this voluntary oral intake protocol to chronic treatments may depend on the experimental model and/or the scientific hypothesis.

These important aspects related with the voluntary oral intake protocol were not described in the paper because we had already discussed them in detail in a previous publication [Teixeira-Santos *et al*., 2021], as cited in the *Experimental design and drug administration* subsection. However, we acknowledge the pertinence of addressing the reviewer’s questions, and, thus, we have now added a sentence referring the readers to our previous publication, underscoring the relevant information that can be found there (lines 136-138).

“The Mouse” - Animal Care and Use Committee from Johns Hopkins University. http://web.jhu.edu/animalcare/procedures/mouse.html, last access: 28/03/2023

Teixeira-Santos L, Albino-Teixeira A, Pinho D. An alternative method for oral drug administration by voluntary intake in male and female mice. *Lab Anim.* 2021;55(1):76-80. doi: 10.1177/0023677220950782.

*2) In this work, the Authors focus on spinal cord changes upon SNI but performed systemic treatment with MaR1 rather than local via intrathecal injection. How do Authors propose MaR1 is reducing glial cell activation? Is this effect indirect or they propose that MaR1 is able to cross BBB? An explanation about how they envision peripheral treatment with MaR1 on the spinal changes is missing.*

Microglia and astrocytes are known to express several SPM receptors and to be susceptible to the effects of different SPMs, indicating that pro-resolution pathways might be critical for the modulation of glial cells’ activation [Tiberi *et al*., 2021]. Taking into consideration that the effects of MaR1 intrathecal treatment have been previously evaluated in NP models [Gao *et al*., 2018; Wang *et al*., 2020; Wei *et al*., 2022], we aimed to investigate whether a systemic treatment also induces spinal cord changes, namely in glial cells’ activation.

Accumulating evidence has been suggesting that MaR1 and other SPMs are able to cross the BBB. Indeed, SPMs administered through different systemic routes have shown to ameliorate inflammation within the CNS (“neuroinflammation”) in numerous models of neurological/neuroimmune diseases, namely by targeting glial cells (as extensively reviewed in Tiberi *et al*., 2021 and Valente *et al*., 2022). In particular, the oral route was used to administer RvD1 in a mouse model of multiple sclerosis and have shown to reduce the pro-inflammatory environment in the CNS and induce an M2 phenotype of monocytes/macrophages and resident brain microglial cells [Poisson *et al.*, 2015]. Furthermore, systemic MaR1 treatments (i.v. or i.p.) have resulted in attenuation of neuroinflammation in models of spinal cord injury and experimental autoimmune encephalomyelitis [Francos-Quijorna *et al.*, 2017; Sánchez-Fernández *et al*., 2022].

SPMs are small lipophilic molecules, which further supports the plausibility of this ability, similarly to what occurs for DHA and EPA (the SPMs’ precursors), which can cross the BBB through simple diffusion [Ouellet *et al*., 2009]. Also, MaR1 has shown ability to maintain integrity/repair dysfunction of the BBB [Yang *et al*., 2022; Li *et al.*, 2022]. Therefore, taking all the evidence into account, we hypothesize that MaR1 may be able to cross the BBB, as supported by other authors. On the other hand, we cannot exclude other possibilities. For example, orally administered RvD1 has been shown to elevate plasma RvD1 concentrations [Recchiuti *et al*., 2014; Mottola *et al.*, 2020]. Increased levels of MaR1 may promote the synthesis of MaR1 itself through enhancement of the MaR1/RORα/12-LOX circuit [Wang *et al.*, 2023]. Thus, indirect actions may also contribute to the observed effects in the CNS, which may reflect putative beneficial effects of MaR1 at the periphery (e.g. anti-inflammatory, anti-oxidative stress, and anti-apoptotic actions, promotion of cell survival, DNA repair, and tissue regeneration).

As suggested by the reviewer, in addition to the evidence that was already mentioned, we have further discussed the abovementioned evidence and hypotheses (lines 610-611, 618-619). We have also underscored the need to clarify those mechanisms in section *On other limitations of the study and future directions* (lines 674-676).

Francos-Quijorna I, Santos-Nogueira E, Gronert K, et al. Maresin 1 Promotes Inflammatory Resolution, Neuroprotection, and Functional Neurological Recovery After Spinal Cord Injury. J Neurosci. 2017;37(48):11731-11743. doi:10.1523/JNEUROSCI.1395-17.2017

Gao J, Tang C, Tai LW, et al. Pro-resolving mediator maresin 1 ameliorates pain hypersensitivity in a rat spinal nerve ligation model of neuropathic pain. *J Pain Res*. 2018;11:1511-1519. doi:10.2147/JPR.S160779

Li T, Zheng J, Wang Z, et al. Maresin 1 improves cognitive decline and ameliorates inflammation and blood-brain barrier damage in rats with chronic cerebral hypoperfusion. Brain Res. 2022;1788:147936. doi:10.1016/j.brainres.2022.147936

Mottola G, Werlin EC, Wu B, et al. Oral Resolvin D1 attenuates early inflammation but not intimal hyperplasia in a rat carotid angioplasty model. Prostaglandins Other Lipid Mediat. 2020;146:106401. doi:10.1016/j.prostaglandins.2019.106401

Ouellet M, Emond V, Chen CT, et al. Diffusion of docosahexaenoic and eicosapentaenoic acids through the blood-brain barrier: An in situ cerebral perfusion study. Neurochem Int. 2009;55(7):476-482. doi:10.1016/j.neuint.2009.04.018

Poisson LM, Suhail H, Singh J, et al. Untargeted Plasma Metabolomics Identifies Endogenous Metabolite with Drug-like Properties in Chronic Animal Model of Multiple Sclerosis. J Biol Chem. 2015;290(52):30697-30712. doi:10.1074/jbc.M115.679068

Recchiuti A, Codagnone M, Pierdomenico AM, et al. Immunoresolving actions of oral resolvin D1 include selective regulation of the transcription machinery in resolution-phase mouse macrophages. FASEB J. 2014;28(7):3090-3102. doi:10.1096/fj.13-248393

Sánchez-Fernández A, Zandee S, Mastrogiovanni M, et al. Administration of Maresin-1 ameliorates the physiopathology of experimental autoimmune encephalomyelitis. J Neuroinflammation. 2022;19(1):27. doi:10.1186/s12974-022-02386-1

Tiberi M, Chiurchiù V. Specialized Pro-resolving Lipid Mediators and Glial Cells: Emerging Candidates for Brain Homeostasis and Repair. Front Cell Neurosci. 2021;15:673549. doi:10.3389/fncel.2021.673549

Valente M, Dentoni M, Bellizzi F, Kuris F, Gigli GL. Specialized Pro-Resolving Mediators in Neuroinflammation: Overview of Studies and Perspectives of Clinical Applications. Molecules. 2022;27(15):4836. doi:10.3390/molecules27154836

Wang YH, Li Y, Wang JN, et al. Maresin 1 Attenuates Radicular Pain Through the Inhibition of NLRP3 Inflammasome-Induced Pyroptosis via NF-κB Signaling. Front Neurosci. 2020;14:831. doi:10.3389/fnins.2020.00831

Wang X, Botchway BOA, Zhang Y, Huang M, Liu X. Maresin1 can be a potential therapeutic target for nerve injury. Biomed Pharmacother. 2023;161:114466. doi:10.1016/j.biopha.2023.114466

Wei J, Su W, Zhao Y, et al. Maresin 1 promotes nerve regeneration and alleviates neuropathic pain after nerve injury. J Neuroinflammation. 2022;19(1):32. doi:10.1186/s12974-022-02405-1

Yang T, Xu G, Newton PT, et al. Maresin 1 attenuates neuroinflammation in a mouse model of perioperative neurocognitive disorders. Br J Anaesth. 2019;122(3):350-360. doi:10.1016/j.bja.2018.10.062

*3) How do Authors explain increase in M-CSF induced by MaR1? Do they see resolution macrophages infiltrating the spinal cord upon treatment?*

M-CSF exerts its actions by activating its receptor, CSFR, which then undergoes dimerization and phosphorylation, thereby inducing the activation of multiple signaling pathways [Tarale *et al*., 2022]. Recently, we have reported increased M-CSF values in male SNI-mice treated with a NADPH oxidase 2(NOX2)-selective inhibitor as compared to their vehicle-treated controls [Teixeira-Santos *et al*., 2023]. Indeed, NOX-dependent generation of reactive oxygen species (ROS) was reported to be selectively required for M-CSF-induced activation of CSFR and specific pathways [Choi *et al*., 2011], further supporting an important relationship between CSFR signaling and redox status [Soto-Diaz *et al*., 2021]. As we have previously discussed in a Review article [Teixeira-Santos *et al*., 2020], SPMs, including MaR1, are known to modulate ROS production through several mechanisms, such as NOX inhibition [Sun *et al*., 2017; Gu *et al*., 2018; Li *et al*., 2021]. Therefore, as we have now further discussed in *On neuroinflammatory mechanisms underlying MaR1 actions* section (lines 657-662), taking into consideration both of our reported observations, we hypothesize that the drug-induced increase in M-CSF values is associated with NOX inhibition/ROS modulation, and those drug-induced effects might have triggered a counterregulatory/compensatory mechanism or a rebound effect in males after treatment completion (lines 653-657).

We do not have data on macrophages’ infiltration into the spinal cord upon treatment, although we acknowledge its relevance. We have added the assessment of macrophage responses as a future direction, in section *On other limitations of the study and future directions* (lines 671-674).

Choi HK, Kim TH, Jhon GJ, Lee SY. Reactive oxygen species regulate M-CSF-induced monocyte/macrophage proliferation through SHP1 oxidation. Cell Signal. 2011;23(10):1633-1639. doi:10.1016/j.cellsig.2011.05.017

Gu J, Luo L, Wang Q, et al. Maresin 1 attenuates mitochondrial dysfunction through the ALX/cAMP/ROS pathway in the cecal ligation and puncture mouse model and sepsis patients. Lab Invest. 2018;98(6):715-733. doi:10.1038/s41374-018-0031-x

Leuti A, Maccarrone M, Chiurchiù V. Proresolving Lipid Mediators: Endogenous Modulators of Oxidative Stress [published correction appears in Oxid Med Cell Longev. 2019 Dec 12;2019:1759464]. Oxid Med Cell Longev. 2019;2019:8107265. Published 2019 Jun 18. doi:10.1155/2019/8107265

Li J, Zhang Z, Wang L, et al. Maresin 1 Attenuates Lipopolysaccharide-Induced Acute Kidney Injury via Inhibiting NOX4/ROS/NF-κB Pathway. Front Pharmacol. 2021;12:782660. doi:10.3389/fphar.2021.782660

Soto-Diaz K, Vailati-Riboni M, Louie AY, et al. Treatment With the CSF1R Antagonist GW2580, Sensitizes Microglia to Reactive Oxygen Species. Front Immunol. 2021;12:734349. Published 2021 Nov 26. doi:10.3389/fimmu.2021.734349

Sun Q, Wu Y, Zhao F, Wang J. Maresin 1 Ameliorates Lung Ischemia/Reperfusion Injury by Suppressing Oxidative Stress via Activation of the Nrf-2-Mediated HO-1 Signaling Pathway. Oxid Med Cell Longev. 2017;2017:9634803. doi:10.1155/2017/9634803

Tarale P, Alam MM. Colony-stimulating factor 1 receptor signaling in the central nervous system and the potential of its pharmacological inhibitors to halt the progression of neurological disorders. Inflammopharmacology. 2022;30(3):821-842. doi:10.1007/s10787-022-00958-4

Teixeira-Santos L, Veríssimo E, Martins S, Sousa T, Albino-Teixeira A, Pinho D. Effects of NADPH Oxidase Isoform-2 (NOX2) Inhibition on Behavioral Responses and Neuroinflammation in a Mouse Model of Neuropathic Pain. Biomedicines. 2023;11(2):416. Published 2023 Jan 31. doi:10.3390/biomedicines11020416

Teixeira-Santos L, Albino-Teixeira A, Pinho D. Neuroinflammation, oxidative stress and their interplay in neuropathic pain: Focus on specialized pro-resolving mediators and NADPH oxidase inhibitors as potential therapeutic strategies. Pharmacol Res. 2020;162:105280. doi:10.1016/j.phrs.2020.105280

*4) It is not clear to me the rationale for performing treatment only from day 3 to 5 (based on schematic provided in figure 1). While the Authors see that 3 days of treatment is enough to reduce pain long after treatment is done, they seem to not see major changes in the spinal cord. Do the Authors have data on the effect of longer treatment protocol with MaR1 (3 to 11, for example).*

Bourquin *et al.*, the authors who developed the SNI model in the mouse, have described that mice develop mechanical hypersensitivity 3 days after the surgery [Bourquin *et al*., 2006]. Also, previous studies have reported that microglial activation begins to increase 3 days after peripheral nerve injury [Zhang *et al*., 2006; Scholz, *et al*., 2008]. Therefore, as explained in lines 147-152, choosing to start MaR1 administration at post-surgical day 3 aimed to increase the translational potential of our findings, by beginning the treatment after the initial development of injury-induced inflammatory processes and NP development (rather than in a prophylactic manner). Furthermore, a previous study had reported that, in adult male rats with spinal nerve ligation, daily intrathecal administration of MaR1 on postsurgical days 3, 4 and 5 attenuated pain hypersensitivity as well as neuroinflammation (e.g., decreased spinal levels of the glial markers GFAP and Iba-1, one week after surgery) [Gao *et al*., 2018]. Since we aimed to extend the therapeutic potential of MaR1 in NP to a systemic route and to both sexes, we decided to follow a similar treatment scheme, which, furthermore, has been used by other authors evaluating pharmacological treatments for NP, based on a similar rationale [Oh *et al*., 2015].

This treatment scheme was a starting point for the assessment of the therapeutic potential of a systemic MaR1 treatment in males and females. We acknowledge the importance of evaluating other schemes, including longer treatment protocols (such as the one suggested by the reviewer), and have now included that in section *On other limitations of the study and future directions* (line 676).

Gao J, Tang C, Tai LW, et al. Pro-resolving mediator maresin 1 ameliorates pain hypersensitivity in a rat spinal nerve ligation model of neuropathic pain. J Pain Res. 2018;11:1511-1519. doi:10.2147/JPR.S160779

Oh SH, So HJ, Lee HY, Lim KJ, Yoon MH, Jung KT. Urinary trypsin inhibitor attenuates the development of neuropathic pain following spinal nerve ligation. Neurosci Lett. 2015;590:150-155. doi:10.1016/j.neulet.2015.01.070

*5) Based on the 3-day treatment protocol, it would be interesting to see the effect of MaR1 on the spinal cord cytokine levels and glial cell markers at 7-day timepoint as well.*

*If possible, it would be also interesting to see whether they see any difference in c-FOS is there, CGRP, and TRPV1 staining for example.*

Indeed, assessing biomarkers (the ones that we have already used and others, such as the ones indicated by the reviewer), namely at an earlier stage, would be important to better understand the mechanisms involved and the evolution of treatment effects. Future studies will address that. In this paper, we focus on glial cell markers assessed some days after treatment completion, as a starting point to evaluate the putative translational therapeutic potential of systemically administered MaR1 in both males and females, which we consider as major strengths of the present work. Furthermore, we have focused on the behavioral assessment of both sensory and affective components of pain.

6) Fig 4: Do Authors see sex-specific modulation of the glial cell markers? Does MaR1 reduce that?

No statistically significant sex differences were found in the modulation of glial cell markers:

**Iba-1**

*Sex*: F(1, 14) = 0.64, P = 0.432; *Treatment × Sex* interaction: (F(1, 14) = 0.43, P = 0.523)

**GFAP**

*Sex*: F(1, 14) = 0.94, P = 0.350; *Treatment × Sex* interaction: (F(1, 14) = 1.24, P = 0.285)

Thus, for immunofluorescence analyzes, and according to what we had previously decided to do if no *Sex* main effects or interactions were detected (lines 327-328), data from males and females within the same treatment group were pooled (lines 446-447).

*7) SNI often induces changes in toe spread. Do Authors see any difference in that parameter? Does MaR1 have an effect on that?*

The evaluation of the distance from the first to the fifth toe (“toe spread”) may be a useful tool for evaluating the extent of injury to the sciatic nerve and the reversal of the functional deficit after pharmacological treatments. No difference between groups was apparent through direct observation of mice’s paw. However, we did not have access to appropriate systems/equipment intended for use in mice, and we did not consider alternative methods such as the “ink method”, since they have been associated with technical difficulties (smeared footprints, surgery-induced contractures) and may stress the animals, which, consequently, would increase the variability of measurements [Varejão *et al*., 2001; Baptista *et al*., 2007]. Moreover, compared to rats, the smaller size of mice’s paw complicates the assessment [Inserra *et al*., 1998].

Baptista AF, Gomes JR, Oliveira JT, Santos SM, Vannier-Santos MA, Martinez AM. A new approach to assess function after sciatic nerve lesion in the mouse - adaptation of the sciatic static index. J Neurosci Methods. 2007;161(2):259-264. doi:10.1016/j.jneumeth.2006.11.016

Inserra MM, Bloch DA, Terris DJ. Functional indices for sciatic, peroneal, and posterior tibial nerve lesions in the mouse. Microsurgery. 1998;18(2):119-124. doi:10.1002/(sici)1098-2752(1998)18:2<119::aid-micr10>3.0.co;2-0

Varejão AS, Meek MF, Ferreira AJ, Patrício JA, Cabrita AM. Functional evaluation of peripheral nerve regeneration in the rat: walking track analysis. J Neurosci Methods. 2001;108(1):1-9. doi:10.1016/s0165-0270(01)00378-8

**Reviewer #3:**

*In the present manuscript, Teixeira-Santos et al studied the antinociceptive properties of MaR1 in a mouse model of SNI. Although several studies have already addressed this issue, the novelty of the present work relies of the fact that the authors have assessed whether the therapeutic properties of this bioactive lipid are sex dependent. Moreover, they also assess the effectivity of MaR1 when administered orally. They found that oral delivery of MaR1 attenuates pain responses in male and female mice and that these results were associated to the ability of this SPM in reducing astrocyte and microglia response in the dorsal horn of the spinal cord.*

*The experiments are well conducted, and the manuscript is interesting for the field.*

*Concerns*

*- Did the author assess macrophage response in the DRG? This information would be of high interest.*

We did not assess it, because we have decided to focus this work on spinal neuroinflammation (and pain-related behavior). Nevertheless, we agree with the reviewer regarding the high interest of assessing macrophage response in the DRG in the context of this work, since DRGs, and DRG macrophages in particular [Yu *et al*., 2020], play a critical role in NP initiation and development [Krames, 2014; Finnerup *et al*., 2021], the SPM MaR1 is synthesized from DHA by macrophages [Dalli and Serhan, 2017], and SPMs are both regulators of macrophage responses and effectors in macrophage-mediated responses [Dalli and Serhan, 2017]. On the other hand, our results on M-CSF also reinforce that interest - Xiaobing *et al.*, 2021 have recently stated that M-CSF “redefines the collaborative contribution of spinal microglia and DRG macrophages to neuropathic pain”. Indeed, it is known that invading macrophages contribute for the sensitization of DRG sensory neurons, which then release M-CSF as a signal to communicate with and activate microglia in the spinal cord [Finnerup *et al*., 2021].

Therefore, we have added the assessment of macrophage response in the DRG as a future direction, in section *On other limitations of the study and future directions* (lines 671-674).

Dalli J, Serhan CN. Pro-Resolving Mediators in Regulating and Conferring Macrophage Function. Front Immunol. 2017;8:1400. doi:10.3389/fimmu.2017.01400

Finnerup NB, Kuner R, Jensen TS. Neuropathic Pain: From Mechanisms to Treatment. Physiol Rev. 2021;101(1):259-301. doi:10.1152/physrev.00045.2019

Krames ES. The role of the dorsal root ganglion in the development of neuropathic pain. Pain Med. 2014;15(10):1669-1685. doi:10.1111/pme.12413

Yu X, Liu H, Hamel KA, et al. Dorsal root ganglion macrophages contribute to both the initiation and persistence of neuropathic pain. Nat Commun. 2020;11(1):264. doi:10.1038/s41467-019-13839-2

Yu X, Basbaum A, Guan Z. Contribution of colony-stimulating factor 1 to neuropathic pain. Pain Rep. 2021;6(1):e883. doi:10.1097/PR9.0000000000000883

*- Please show high magnification images of astrocytes and microglia*

We have quantified the fluorescent intensity (mean gray value) of Iba-1 and GFAP in the dorsal horn as the method of choice for the glial activation’ analysis, similar to what has been reported by several other authors (e.g., Hathway et al., 2009; Adães et al., 2017; Zhang et al., 2012; Zucoloto et al., 2019). Moreover, in our pilot experiment (following the same protocol), Sham animals showed an intensity ratio around 1 (*i.e.*, similar expression on both the contralateral and ipsilateral sides), which indicates the validity of our approach. Therefore, in Figure 4, we have presented representative images of the immunofluorescence staining used in our analysis.

In an attempt to address the reviewers’ request, and acknowledging that higher magnification images would enable a better visualization of astrocytes and microglia morphology, we tried to take high magnification photos of the same sections presented in Figure 4, which we herein present.

Representative images of the immunofluorescence staining with Iba-1 and GFAP in the spinal cord of mice. Scale bar: 100 μm (larger images) and 20 μm (smaller images)

Unfortunately, considering that the original experiment was conducted in 2020 (and although we have all the slides properly stored), we think that the images no longer have sufficient quality to be presented in the article.

Alternatively, in order to enable to zoom in the individual images, we have uploaded the original microscopy files as supporting information. These image files have .zvi extensions, and can be opened by the open-source software Fiji, for example.

Adães S, Almeida L, Potes CS, Ferreira AR, Castro-Lopes JM, Ferreira-Gomes J, Neto FL. Glial activation in the collagenase model of nociception associated with osteoarthritis. Mol Pain. 2017;13:1744806916688219. doi:10.1177/1744806916688219

Hathway GJ, Vega-Avelaira D, Moss A, Ingram R, Fitzgerald M. Brief, low frequency stimulation of rat peripheral C-fibres evokes prolonged microglial-induced central sensitization in adults but not in neonates. Pain. 2009;144(1-2):110-8. doi:10.1016/j.pain.2009.03.022

Zhang H, Yoon SY, Zhang H, Dougherty PM. Evidence that spinal astrocytes but not microglia contribute to the pathogenesis of Paclitaxel-induced painful neuropathy. J Pain. 2012;13(3):293-303. doi: 10.1016/j.jpain.2011.12.002.

Zucoloto AZ, Manchope MF, Borghi SM, Dos Santos TS, Fattori V, Badaro-Garcia S, Camilios-Neto D, Casagrande R, Verri WA Jr. Probucol Ameliorates Complete Freund's Adjuvant-Induced Hyperalgesia by Targeting Peripheral and Spinal Cord Inflammation. Inflammation. 2019; 42(4):1474-1490. doi:10.1007/s10753-019-01011-3

*- Please indicate whether error bars in the figures represent SEM or SD*

Results are expressed as predicted means, with error bars corresponding to 95% confidence interval (CI). This information is stated in lines 334 (*Statistical Analyses* section), as well as in the caption of each figure (Line 358 – Fig 2; Lines 385 and 387 – Fig 3; Line 458 – Fig 4; Line 480 – Fig 5).
