# Supplementary material for: The pro-resolving lipid mediator Maresin 1 ameliorates pain responses and neuroinflammation in the spared nerve injury-induced neuropathic pain: A study in male and female mice
Source: PLoS One. 2023 Jun 22;18(6):e0287392. doi: 10.1371/journal.pone.0287392 (PMC10286986; doi:10.1371/journal.pone.0287392)
Supplement: Attachment — Submitted filename: PlosOne_MaR1_Response-to-Reviewers_(2).docx [file pone.0287392.s006.docx]

**Response to Reviewers**

We thank the reviewers for their valuable time and thoughtful feedback. Please find below our response. For editor’s and reviewers’ convenience, both the complete replies to reviewers and a **track-changes version of the manuscript** have been uploaded. In the replies to reviewers, mentions to “line numbers” refer to the final manuscript, rather than the “track-changes version of the manuscript”.

**Reviewer #2**

**Reviewer’s Comment**

“While the MS has improved after revision, this Reviewer understands that more mechanistic details are missing to explain the analgesic effect they observed. Also, data on the on the effect of the longer treatment protocol with MaR1 is required.”

**Response**

Although we acknowledge the pertinence of the Reviewer’s comment, we consider that the observations described in our article are important to report at this phase of our project, since:

- It is the first time that Maresin 1 was used in a NP model through an oral route and in both sexes;
- MaR1 treatment was administered in an already installed peripheral NP model;
- We have shown antinociceptive effects of MaR1 treatment that extend for several days beyond the treatment time-frame;
- The antinociceptive effects produced are associated with downregulation of Iba-1 and GFAP in both sexes, while possibly involving modulation of M-CSF action in males.

Moreover, some of our protocols and observations (e.g. PEAP assay, M-CSF values) open a range of possibilities and questions that could be of interest in other fields as well.

Addressing the Reviewer concern on mechanistic details to explain the analgesic effect of MaR1, we have added more details about the MaR1’s mechanisms of action that have been reported by other authors in different models of pain (lines 615-624, 684-687). In fact, there are already several lines of evidence that help to explain the antinociceptive effects of MaR1 and specialized pro-resolving mediators (SPMs) in general, as has been extensively reviewed by many authors, including anti-inflammatory, neuroprotective and pro-resolutive actions, through regulation of different signal pathways in the periphery and in the nervous system (Teixeira-Santos *et al*., 2020; Leuti *et al*., 2021; Wang *et al*., 2023).

As for the concern pertaining the effect of longer treatment protocol with MaR1, we must stress that both treatment duration and behavioral pain assessment/tissue collection timeline were carefully considered beforehand, as explained in the Methods section (lines 148-163):

*“The treatment scheme selected aimed to increase the translational potential of our findings, by evaluating the effects of MaR1 treatment when administered after activation and initial development of SNI-induced inflammatory processes and of mechanical hypersensitivity [32,33], and was similar to the one used by Gao et al., who reported that daily intrathecal administration of MaR1 on postsurgical days 3, 4 and 5 attenuated pain hypersensitivity and neuroinflammation in a NP rat model [20]. (…) The sensory-discriminative component of pain was evaluated with the von Frey test, before surgery (baseline), and on days 7 and 11 post-SNI, whereas the place escape/avoidance paradigm (PEAP) test was performed only once, on day 11, for the evaluation of the affective-motivational component of pain. Each mouse was subjected to either von Frey or PEAP protocols. Euthanasia and tissue collection were performed on day 12 (Fig 1). The behavioral pain assessment and tissue collection timeline was designed to evaluate the ability of a “short” MaR1 treatment to both reverse the already initiated SNI-induced inflammatory processes and mechanical hypersensitivity, and prevent the NP chronification.”*

Please see also the reformulated discussion on the novelty and translational potential of our results (lines 503-517):

*“Our results expand previously reported data showing antinociceptive actions of MaR1 treatment on different peripheral NP models. In most of the referred studies, MaR1 was intrathecally administered – non-compressive lumbar disk herniation (daily MaR1 administration during the first 3 postsurgical days; daily behavioral testing for mechanical and thermal hypersensitivity up to postsurgical day 7 [22,23]); spinal nerve ligation (daily MaR1 administration from postsurgical days 3 to 5; daily hypersensitivity assessment up to postsurgical day 7 [20]); chronic constriction injury (single MaR1 injection 1 week after injury; hypersensitivity assessment up to 24h post-MaR1 dosing [21]) – although there is also one publication, by Serhan et al., reporting the antinociceptive effects of systemic MaR1 treatment in the vincristine-induced model of peripheral NP (i.p. injection of MaR1 shortly before administration of the chemotherapeutic agent vincristine reduced mechanical hypersensitivity between days 1 and 14 [15]). Our results not only further demonstrate the effectiveness of systemically administered MaR1 in a peripheral NP model, but specifically show for the first time that MaR1 administered through a voluntary oral protocol can ameliorate pain hypersensitivity in a NP model already installed, and maintain those antinociceptive effects for at least for six days beyond the treatment time window.”*

Nevertheless, we agree that other protocols could be investigated – and are, indeed, necessary to advance the knowledge on this putative new therapeutic approach to chronic pain.

In the section *On other limitations of the study and future directions*, we acknowledge that our study has a number of limitations, and we list them (lines 681-700). Furthermore, we suggest several future directions for research in this area and have underscored the need to replicate and clarify our results. Unfortunately, due to time and resource constraints, we will not be able to perform the additional experiments within an adequate timeframe for publication in this review process.

Nevertheless, upon further reviewing the literature on this topic, we still believe that we have contributed to add further and relevant knowledge about the effects of MaR1 (and SPMs in general). The novelty and the focus of our paper lie on extending the therapeutic potential of MaR1 in neuropathic pain to both sexes and to an oral route, which had not been investigated before. Also, we have evaluated both the sensory and affective components of pain, as well as relevant spinal neuroinflammatory parameters, namely microglial and astrocytic activation, and concentration of both pro- and anti-inflammatory cytokines. For that, we have used reliable techniques, *i.e.* immunofluorescence microscopy and multiplex immunoassays, which indicated that MaR1 attenuated SNI-induced spinal microglial and astrocytic activation in male and female mice and that M-CSF concentrations were marginally higher in MaR1-treated SNI males, on day 12. We have also been able to dissect the time-frames of the antinociceptive and anti-glial activation actions of MaR1 from those of its downregulatory effects on pro-inflammatory cytokines, as discussed on lines 642-650. Furthermore, we have tried to contribute to the chronic pain field as a whole, by developing a voluntary oral intake protocol and providing new insights into the Place Escape/Avoidance Paradigm (PEAP) behavioral test, since ours is one of the first studies that evaluated the behavior of female rodents in this paradigm. Indeed, we propose that the PEAP test may not be appropriate to ascertain the emotional pain behavior in female C57BL/6J mice with SNI-induced NP and that measures more focused on changes of behavior throughout the test period should be analyzed instead of/in addition to “total time in light”.

We believe that all of these are major factors in terms of translational relevance and significantly strengthen our manuscript.

We hope that these arguments address the Reviewer’s concerns, and they may consider our manuscript acceptable for publication.

Teixeira-Santos L, Albino-Teixeira A, Pinho D. Neuroinflammation, oxidative stress and their interplay in neuropathic pain: Focus on specialized pro-resolving mediators and NADPH oxidase inhibitors as potential therapeutic strategies. *Pharmacol Res.* 2020;162:105280. doi: 10.1016/j.phrs.2020.105280

Leuti A, Fava M, Pellegrini N, Maccarrone M. Role of Specialized Pro-Resolving Mediators in Neuropathic Pain. *Front Pharmacol.* 2021;12. doi: 10.3389/fphar.2021.717993

Wang X, Botchway BOA, Zhang Y, Huang M, Liu X. Maresin1 can be a potential therapeutic target for nerve injury. *Biomed Pharmacother*. 2023;161:114466. Epub 2023/03/05. doi: 10.1016/j.biopha.2023.114466
